# Inhibition of Non-Homologous End Joining Repair Impairs Pancreatic Cancer Growth and Enhances Radiation Response

**DOI:** 10.1371/journal.pone.0039588

**Published:** 2012-06-18

**Authors:** Ying-Hua Li, Xiaoxu Wang, Yunfeng Pan, Dong-Hyun Lee, Dipanjan Chowdhury, Alec C. Kimmelman

**Affiliations:** Division of Genomic Stability and DNA Repair, Department of Radiation Oncology, Dana-Farber Cancer Institute, Harvard Medical School, Boston, Massachusetts, United States of America; Technische Universität München, Germany

## Abstract

Pancreatic ductal adenocarcinoma (PDAC) is amongst the deadliest of human cancers, due to its late diagnosis as well as its intense resistance to currently available therapeutics. To identify mechanisms as to why PDAC are refractory to DNA damaging cytoxic chemotherapy and radiation, we performed a global interrogation of the DNA damage response of PDAC. We find that PDAC cells generally harbor high levels of spontaneous DNA damage. Inhibition of Non-Homologous End Joining (NHEJ) repair either pharmacologically or by RNAi resulted in a further accumulation of DNA damage, inhibition of growth, and ultimately apoptosis even in the absence of exogenous DNA damaging agents. In response to radiation, PDAC cells rely on the NHEJ pathway to rapidly repair DNA double strand breaks. Mechanistically, when NHEJ is inhibited there is a compensatory increase in Homologous Recombination (HR). Despite this upregulation of HR, DNA damage persists and cells are significantly more sensitive to radiation. Together, these findings support the incorporation of NHEJ inhibition into PDAC therapeutic approaches, either alone, or in combination with DNA damaging therapies such as radiation.

## Introduction

Pancreatic ductal adenocarcinoma (PDAC) remains the fourth leading cause of cancer mortality in the United States [1], and is characterized by an intense resistance to chemotherapy and ionizing radiation (IR). Because of this, the majority of patients will succumb to their disease in less than one year and novel therapeutic approaches are clearly needed. Genomic instability is one of the hallmarks of cancer [2] and consistent with this we and others have shown that pancreatic cancers display extremely high levels of genomic alterations [3]. Furthermore, pancreatic cancers are profoundly resistant to DNA damaging therapies such as cytotoxic chemotherapy and radiation [4]. However, the biological significance of genomic instability in this disease and how this may impact the response to DNA damaging therapies is relatively unexplored.

Double stranded breaks (DSBs), induced by radiation or other DNA damaging agents, are believed to be the most hazardous DNA lesions that threaten cellular survival. In response to ionizing radiation, DSBs are detected by the Mre11–Rad50–Nbs1 complex (MRN complex) and Ku70/Ku80 complexes which rapidly activate ataxia telangiectasia mutated (ATM) and DNA-PK respectively [5]. Activation of these kinases induces a series of cellular events including phosphorylation of cell cycle checkpoint proteins and the initiation of the DNA repair process. Histone H2AX, an important substrate of ATM and DNA-PK, is phosphorylated on serine 139 (referred to as γH2AX), which forms foci on DSB sites associated with other repair factors [6].

Two major pathways exist to repair DSBs -homologous recombination (HR) and non-homologous end-joining (NHEJ) [7], [8]. HR-directed repair requires an homologous chromosome or a sister chromatid as a template to repair DNA with high fidelity, and therefore it mainly occurs in S- and G2- phases of the cell cycle when the template is available. In contrast to HR, NHEJ repairs DSB by ligation of two DNA ends following DNA end processing. The end processing often leads to loss of nucleotides and makes NHEJ error-prone [9]. NHEJ is active throughout the cell cycle. Therefore, cell cycle stage and the nature of DNA ends are two determinants of repair choices between HR and NHEJ [7], [10]. In addition, DNA-PK activity itself has been implicated in the inhibition of HR [11], [12]. Importantly, cancer cells often show abnormalities in the DNA damage response and defects in DNA repair which may correlate with altered expression of repair proteins. For example, higher expression of the NHEJ proteins, DNA-PK and Ku70/80 has been reported in cancer cell lines [13], [14], [15], [16] However, the DNA damage response and DNA repair in PDAC cells remains relatively unexplored.

Here we investigated the importance of DNA repair in PDAC biology and find that PDAC cells harbor elevated levels of basal DNA damage. Inhibition of NHEJ results in increased DNA damage and ultimately decreased proliferation. In response to NHEJ inhibition, HR is upregulated but cells are unable to repair DNA damage efficiently in response to radiation. This results in increased radiation sensitivity as evidenced by decreased clonogenic survival. Our data implicate NHEJ inhibition as a potential therapeutic approach in PDAC.

## Results

### Basal DNA damage in PDAC

**Figure 1:**
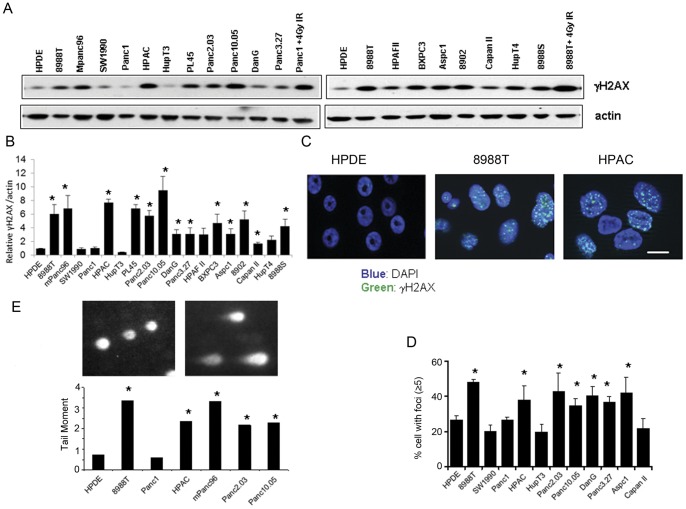
Elevated spontaneous DNA damage in PDAC cells. (A) Western blot analysis of γH2AX in HPDE and 18 PDAC cell lines. Samples from 8988T and Panc1 irradiated at 4Gy were used as positive controls and actin served as the loading control. Experiments were done at least three times. (B) Quantitation of γH2AX expression by densitometry was normalized to actin expression and presented as relative to HPDE. Data is shown from three independent experiments with error bars representing standard deviations. (C) Immunofluoresence for basal γH2AX foci in three representative cell lines (HPDE, 8988T and HPAC). Green: γH2AX; Blue: DAPI (nucleus). Scale bar equals 10 µm. Quantitation was performed in (D) and shown as percentage of cells with more than 5 foci. Experiments were done three times and average values were calculated. Error bars represent standard deviation from three individual experiments. (E) Neutral comet assay was performed to directly assess DNA double strand breaks and the data expressed as tail moment (Tail moment = tail length ×% of DNA in the tail). For all panels, asterisks show a statistically significant increase as compared to HPDE by t-test (p≤0.05).

In an effort to understand why PDAC are profoundly resistant to DNA damaging therapies, such as cytotoxic chemotherapy and radiation therapy, we undertook an effort to understand the DNA damage response and DNA repair in these tumors. As an initial step, basal levels of DNA damage were examined in a collection of 18 PDAC cell lines as well as a non-transformed immortalized human pancreatic ductal cell line (HPDE) [17] as a control. Western blot analysis for γH2AX, a widely used marker for DNA damage, particularly DNA double strand breaks (DSBs) [18], [19] was performed. Strikingly, more than half of the PDAC cell lines showed elevated levels of γH2AX (Figure 1A and 1B) compared to HPDE. To confirm that the elevated levels of γH2AX were not merely due to increased levels of total histones, we normalized the levels of γH2AX to total H2AX in selected cells lines (data not shown) and demonstrated that these lines continued to have elevated γH2AX compared to HPDE cells. As further evidence of the elevated DNA damage in PDAC, γH2AX immunofluorescence was performed in representative PDAC cell lines. These analyses were largely consistent with the western blot data, with foci in PDAC lines typically higher than in HPDE (Figure 1C and 1D). To directly measure DNA damage, neutral comet assays were performed to evaluate the amount of double-strand breaks under basal conditions. Again consistent with the γH2AX data, four of the five PDAC cell lines assayed demonstrated statistically higher tail moments than that in HPDE (Figure 1E), indicating increased DSBs. Taken together, these data indicate that PDAC cells often possess elevated basal levels of DNA damage which may result in activation of a DNA damage response.

### DNA Repair Pathways in PDAC

**Figure 2:**
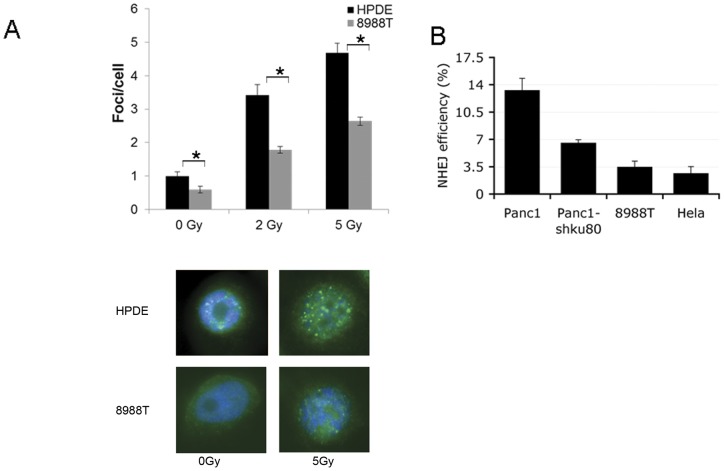
Pancreatic cancers have low levels of HR and are proficient in NHEJ repair. (A) HR in response to increasing doses of radiation (0, 2, and 5 Gy) was measured by Rad51 foci formation and expressed as average foci per cell. Foci are increased in HPDE cells with increasing doses of radiation, while are only minimally changed in 8988T cells. Data are from two independent experiments performed with replicate coverslips with error bars representing standard deviations. Asterisks show a statistically significant difference by t-test (p≤0.05). Representative images are shown below. (B) NHEJ measured by a plasmid luciferase repair assay. Data are normalized for transfection efficiency and then to uncut luciferase. Both Panc1 and 8988T cells show appreciable NHEJ repair. Knockdown of Ku80 markedly decreased NHEJ in Panc1 cells. Error bars represent standard deviations of three independent experiments.

The two major DNA repair pathways for double strand break repair are homologous recombination (HR) and non-homologous end joining repair. HR repair requires an homologous chromosome or a sister chromatid as a template and mainly takes place in S or G2 phase of cell cycle to precisely repair damaged DNA [7]. NHEJ, however, repairs DNA by directly ligating the DNA ends after end processing which often introduces loss of nucleotides and makes NHEJ error-prone [9]. Given the elevated basal levels of DNA damage in PDAC, we assessed the proficiency of these cells for HR and NHEJ. To measure the relative amount of HR, we performed immunofluorescence for Rad51, a critical HR protein which binds to single-stranded DNA overhangs and catalyzes the process of DNA strand exchange. The formation of Rad51 foci is a sensitive and specific indicator of HR [20], [21]. Basal levels of Rad51 foci were low in 8988T PDAC cells as well as in HPDE cells (Fig 2A). While HPDE cells showed a marked increase in Rad51 foci with increasing doses of radiation, 8988T cells showed a significantly lower increase in foci even at 5 Gy of radiation (Fig 2A). Given the minimal levels of HR seen in this PDAC line, we speculated that these cells may rely primarily on NHEJ for repair. To assess NHEJ, we utilized a well characterized luciferase-based plasmid repair assay [22], [23]. In brief, a cut luciferase plasmid (PGL2) is transfected into cells and repair via NHEJ is measured by relative luciferase activity. Both 8988T and Panc1 PDAC lines demonstrated proficiency in NHEJ as demonstrated in Figure 2B.

### DNA-PK is required for PDAC growth

**Figure 3:**
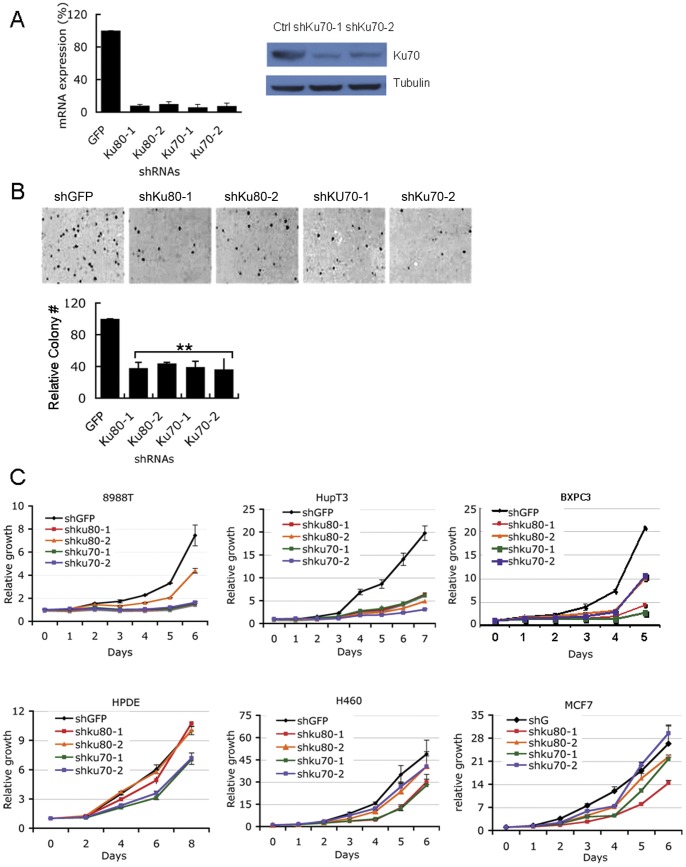
NHEJ is required for PDAC growth (A) Suppression of Ku70 or Ku80 expression in 8988T cells by shRNAs detected by quantitative real-time PCR (left) and western blot (right). (B) Upper panel: representative images of soft agar colony formation of 8988T cells infected with either a control shRNA to GFP or two different shRNAs to Ku70 or Ku80 respectively. Lower panel: quantitation of soft agar colony formation of 8988T cells relative to shGFP infected cells. Results are averages of three independent experiments and error bars represent standard deviations. Two asterisks indicate statistical significance: *P*<0.01 by t-test (C) Growth curve assays were performed to assess the effect of inhibition of NHEJ by Ku70 and Ku80 knockdown on PDAC growth. Note the robust suppression of growth in PDAC cell lines (8988T, HupT3, BXPC3) and only modest effects on other cell lines (MCF7, H460 and HPDE).

**Figure 4:**
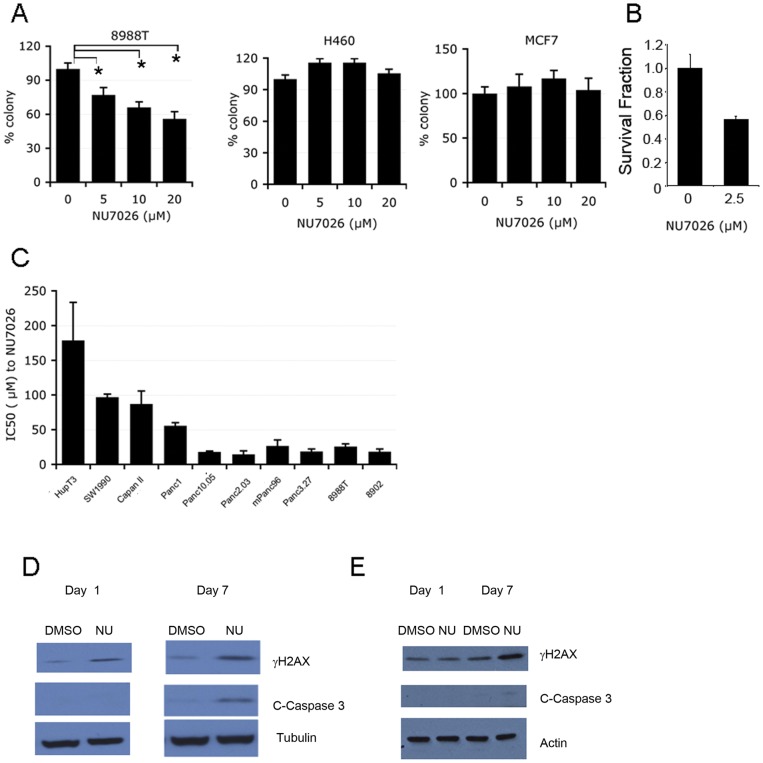
Pharmacologic Inhibition of NHEJ impairs PDAC growth. (A) Soft agar assays in 8988T PDAC cells and tumor cell lines of other histologies (H460 and MCF7) with increasing doses of the DNA-PK inhibitor NU7026 demonstrates the dose-dependent inhibition of anchorage-independent growth in PDAC cells but only minimal effects in the other cell lines assayed. Asterisks show a statistically significant difference by t-test (p≤0.05). (B) Clonogenic survival of 8988T cells treated with NU7026 showing decreased clonogenic growth. (C) IC50s to NU7026 across a larger panel of PDAC cell lines show the majority are sensitive to DNA-PK inhibition. Error bars represent standard deviations from three independent experiments. (D) NU7026 treatment of 8988T cells induced DNA damage and apoptosis measured by γH2AX and cleaved caspase-3 respectively. 8988T cells were treated with NU7026 or DMSO as a control for one day or seven days followed by western blot analysis. Increased DNA damage was seen at the early (Day 1) timepoint with increasing damage and ultimately apoptosis seen on day 7. (E) HPDE cells in comparison show a small increase in γH2AX expression, but very minimal increase in cleaved caspase-3.

Given the results from the DSB repair assays, we next asked whether PDAC cells rely on NHEJ for normal proliferation and growth. We focused on inhibition of DNA-PK, which consists of the Ku70/Ku80 heterodimeric protein and the catalytic subunit, DNAPKcs, and is essential for NHEJ [24]. Using shRNAs, we suppressed expression of the regulatory subunits of DNA-PK, Ku70 and Ku80 in 8988T cells. Both shRNAs produced a robust decrease of the expression of Ku70 or Ku80 in 8988T cells, which was detected by qRT-PCR for Ku70 and Ku80 and by western blot for Ku70 (Figure 3A). Depletion of Ku70 or Ku80 significantly inhibited the anchorage independent growth of 8988T as assessed by softagar assays, as well as proliferation by growth curve assay (Figure 3B and 3C). Suppression of Ku70 or Ku80 also decreased the growth rate of two additional PDAC cell lines, HupT3 and BXPC3 (Figure 3C). In contrast to PDAC, depletion of Ku70 or Ku80 in HPDE, MCF7 (a breast cancer cell line) or H460 (a lung cancer cell line) showed more modest effects on proliferation (Figure 3C). These data suggest that PDAC cells require Ku70, Ku80 for growth. To further analyze the requirement of NHEJ to maintain PDAC growth, a pharmacological DNA-PK inhibitor, NU7026 [25], [26], was used to investigate the involvement of DNA-PK in PDAC growth. NU7026 treatment significantly decreased the anchorage-independent growth of PDAC cells, while the growth of H460 and MCF7 was not affected even at the highest doses (20 µM) (Figure 4A). Additionally, we determined the sensitivities of a collection of PDAC lines to NU7026 by determining the IC50s (Fig 4C). More than half of the lines were sensitive to DNA-PK inhibition. NU7026 also decreased clonogenic survival of 8988T PDAC cells (Figure 4B). Together, the data support that PDAC cells rely on NHEJ DNA repair for growth under basal conditions.

One potential outcome, when damaged DNA is left unrepaired is that cells will eventually undergo apoptosis [27], [28]. To assess the cellular consequences of DNA-PK inhibition on PDAC cells, we looked at markers of DNA damage and apoptosis after short (1 day) or long-term (7 days) treatment with NU7026. Indeed, we found that DNA-PK inhibition for one day caused additional DNA damage analyzed by γH2AX. However, at the seven day timepoint, increased cleaved caspase-3 expression became apparent indicating that apoptosis may be elevated in NU7026 treated cells but not in DMSO treated control cells (Figure 4D). In contrast, treatment of HPDE cells with NU7026 showed increased γH2AX with very minimal cleaved caspase-3 expression (Figure 4E). Thus, inhibition of the NHEJ pathway leads to a further accumulation of DSBs, checkpoint activation and ultimately apoptosis in PDAC cells.

### DSB repair in PDAC after IR is dependent on DNA-PK

**Figure 5:**
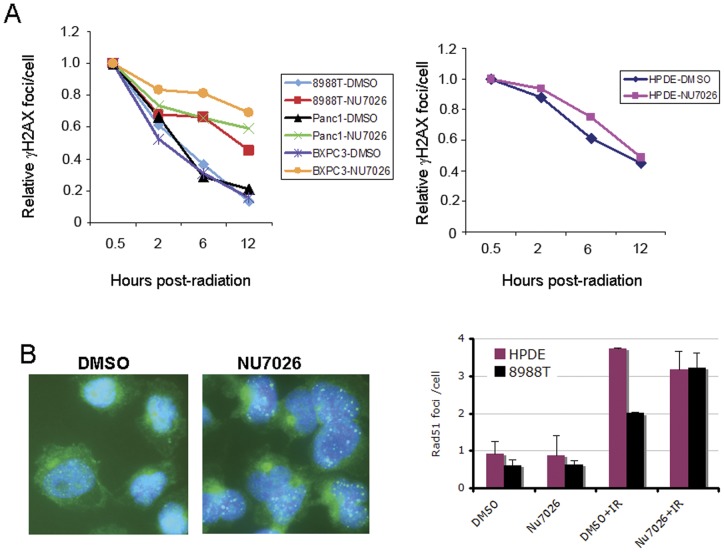
Double Strand Break (DSB) repair in PDAC cells is attenuated by NHEJ inhibition. (A) Repair kinetics of three PDAC cell lines (8988T, Panc1 and BXPC3) were measured by γH2AX staining at various time points after radiation. Data is shown as foci number per cell, normalized to the 30 min timepoint when foci formation was maximal. Each cell line was treated with NU7026 (20 µM) or DMSO. In each line the resolution of foci was substantially delayed with NU7026 treatment. Similar experiments were performed in HPDE cells (right panel). Note that the NU7026 does not attenuate foci resolution in these cells. (B) HR is increased as a compensatory response to NHEJ inhibition in PDAC cells. Rad51 foci formation is markedly increased when DNA-PK activity is inhibited by NU7026. Left panel: Rad51 foci in green and nucleus in blue shown by DAPI staining. Right panel: quantitation of foci per cell at the indicated conditions. Data is shown from two independent experiments with error bars representing standard deviations of the mean.

To further explore the response of PDAC cells to DNA damage, we measured the repair kinetics of three PDAC cell lines following IR by monitoring γH2AX foci at 30 min, 2, 6 and 12 hours after IR with a clinically relevant dose of 2Gy. γH2AX foci peaked at around 30 min after IR. At 12 h post IR, the number of γH2AX foci in all three PDAC cells returned to basal level. We further found that the repair of all three PDAC cell lines was significantly attenuated by inhibition of DNA-PK, indicating that the repair of DSBs in PDAC cells is highly dependent on NHEJ (Figure 5A). Interestingly, inhibition of DNA-PK had only a minimal effect on the repair of HPDE cells (Fig 5A). We next examined how HR repair was impacted by inhibition of NHEJ. As shown previously, HR was lower in 8988T cells even after IR compared to HPDE. However, HR was significantly increased in the presence of NU7026 in 8988T cells after IR (Figure 5B). Despite the apparent compensatory increase in HR in 8988T cells where NHEJ is inhibited, the number of γH2AX foci remains high (Figure 5A), indicating that this is still not sufficient for complete repair.

### NHEJ inhibition sensitizes PDAC to IR

**Figure 6:**
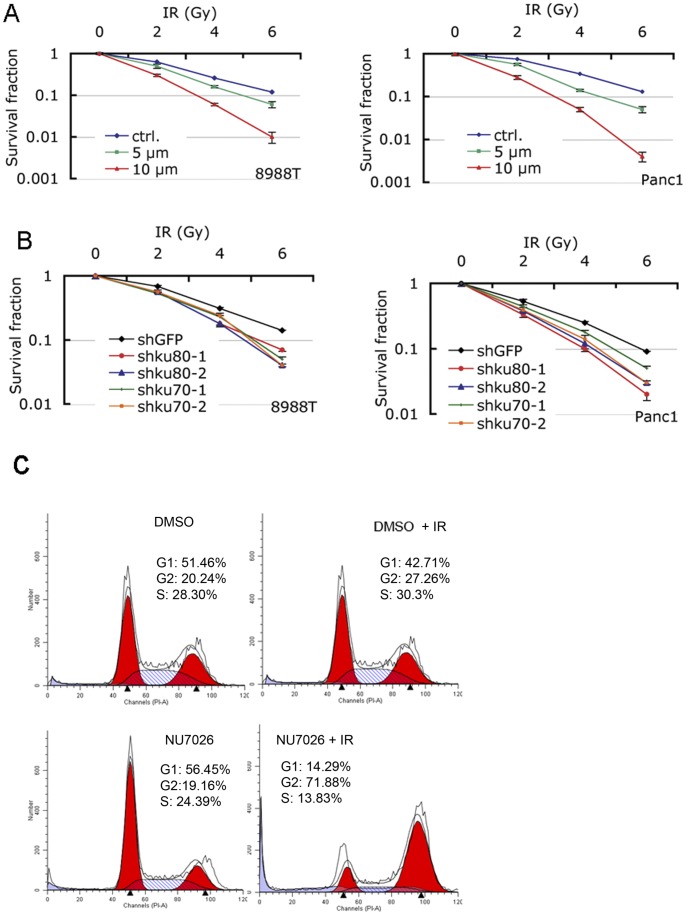
NHEJ inhibition radiosensitizes PDAC cells. (A) Inhibition of DNA-PK with NU7026 radiosensitizes 8988T and Panc1. Cells were plated in 6-well plates in triplicate and treated with DMSO or NU7026 at 20 μM the next day. Cells were subjected to IR after overnight treatment with DMSO or NU7026. After 9 days, cells were fixed, stained, and colonies counted. The surviving fraction was calculated using the plating efficiency. (B) Suppression of Ku70 or Ku80 radiosensitized PDAC cell lines, 8988T and Panc1. 8988T or Panc1 cells were infected with shKu70 or shKu80 and shGFP was used as control. Assays were performed as in (A). (C) Typical cell cycle distribution of 8988T at indicated treatments. 8988T cells were treated with NU7026 or DMSO for two days followed by IR at 2Gy and stained with propidium iodide 24 hours later. Cell cycle was analyzed by flow cytometry. Experiments were repeated three times and shown is the representative result.

Inhibition of NHEJ increases DNA damage leading to decreased growth and apoptosis in PDAC cells, as well as results in the prolonged presence of DNA damage foci following radiation. Therefore, NHEJ inhibition would be expected to enhance the efficacy of radiation. Both 8988T and Panc1 cells showed increased sensitivity to IR when they were treated with NU7026 as shown by decreased clonogenic survival (Figure 6A). Similarly, 8988T and Panc1 cells with Ku70 or Ku80 knockdown were also more sensitive to IR (Figure 6B). Consistent with the decreased clonogenic cell survival, an increased fraction of 8988T cells were arrested at the G2/M phase of the cell cycle when cells were treated with NU7026 and IR (71.88% vs. 27.26% in the irradiated control) compared to control treated or irradiated cells (Figure 6C).

## Discussion

Pancreatic cancers are profoundly resistant to current therapeutic approaches, including those that work via inducing DNA damage such as various cytotoxic chemotherapies and radiation. Thus, understanding the response of these tumors to DNA damage may provide key therapeutic insights. Our work demonstrates that pancreatic cancer cell lines often have elevated levels of basal DNA damage in the absence of exogenous damaging agents. While the exact etiology of the increased basal DNA damage is not known, it is tempting to speculate that the constant genomic instability that these tumors endure [3] may be partially responsible. For example, during the process of genomic amplification, breakage-fusion-bridge cycles occur which result in the formation of continuous transient DSBs [29]. These and other genomic events frequently seen in PDAC, such as chromosomal translocations, may promote a steady state of DNA damage.

Our findings also suggest that NHEJ is the major pathway responsible for DSB repair in pancreatic cancer cells as inhibition of NHEJ abolishes the rapid repair kinetics. Together, the evidence would suggest a scenario where these tumors have developed a reliance on NHEJ to survive continuous genomic instability and the resultant DNA damage that ensues. The selection of DSB repair pathway and the relationship between HR and NHEJ are of great scientific interest and while the exact molecular underpinnings of pathway selection are being worked out, it is likely that at least part of the repair choice is dictated by the cell-cycle, as HR can only function in S/G2/M [7]. We show that HR is quite low in PDAC cells, even in response to IR. However, there is a compensatory increase in HR when NHEJ is inhibited, but, it is insufficient to prevent genotoxic levels of DNA damage.

Additionally, our results are also in line with the recent therapeutic approaches in BRCA1 and 2 mutant tumors using PARP inhibitors. This “synthetic lethality”, where tumors with a single DNA repair pathway such as HR is impaired are sensitive to inhibition of a second repair pathway (e.g. BER), has received much attention [30], [31], [32]. In line with the concept of inhibiting DNA repair pathways as a therapeutic approach, PDAC show a sensitivity to NHEJ inhibitors. One potential explanation is that the elevated basal DNA damage serves to overwhelm the DSB repair making them susceptible to inhibition of NHEJ or other DNA repair pathways.

Lastly, inhibition of NHEJ shows a strong synergy with radiation. This is not unexpected from a mechanistic standpoint, but may have clinical implications in the treatment of the disease. While distant disease is a significant cause of mortality in pancreatic cancer, recent data shows that as many as 30% PDAC deaths are directly attributable to local progression [33]. Unfortunately, surgery is not possible for the majority of these patients and the only other available local therapy, radiation, is ineffective in the majority of cases, even when combined with chemotherapy [34]. Therefore, increasing the sensitivity of these tumors to radiation could have a transformative impact in this population of patients. Indeed, the development of inhibitors to DNA repair proteins is occurring rapidly and many are moving into the clinic as components of cancer treatment [35]. The mechanistic insights identified in this study, provide a compelling molecular rationale to explore the development of such agents for the treatment of pancreatic cancer.

## Materials and Methods

### Cell culture and reagents

The human tumor cell lines were obtained from the American Type Culture Collection or the German Collection of Microorganisms and Cell Cultures. HPDE cells were obtained from M.S. Tsao. [17]. Cells were grown in either DMEM or RPMI supplemented with 10% cosmic calf serum, antibiotics and glutamine. HPDE cells were cultured in keratinocyte serum-free (KSF) medium supplemented by bovine pituitary extract and epidermal growth factor (Gibco). NU7026 (Sigma) was used at 20 μM unless otherwise noted.

### Real-time PCR

The RNA was isolated with TRIzol (Invitrogen), DNase-treated and reverse transcribed to cDNA using MMLV High Performance Reverse Transcriptase (Epicentri) following the manufacturer's instruction. PCR was carried out using the SYBR Green detection reagent (Applied Biosystems) in a Bio-Rad Chromo4 Thermocycler. PCR amplification was performed at 95°C for 2 min followed by 40 cycles of 95°C for 15 sec, 55°C for 15 sec and 72°C for 30 sec. Finally a melting curve was generated from 55°C to 95°C, read every 0.5°C. The primers were as follows: Ku70, forward 5′- AGTCATATTACAAAACCGAGGGC -3′ and reverse 5′- CCTTGGAGGCATCAACCAAAAA -3′ Ku80 forward 5′- CCTTTCTGGTGGGGATCAGTA -3′ and reverse 5′- ACCTGGTTGGATTTTGCTTTCAA -3′.

### IC50 assay

Cells were plated in 96-well plates and treated by serial dilution of NU7026 the next day for 72 h. Cell viability was measured using the Cell-Titer-Glow assay (Promega, G7570) according to the manufacturer's instructions. The IC50 was calculated using a sigmoidal model using BioDataFit 1.

### shRNA transfection

pLKO.1 plasmids containing shRNA sequence for Ku70 and Ku80 were obtained from The RNAi Consortium (sequences available upon request). Lentivirus containing Ku70, Ku80 and GFP control shRNAs were produced in HEK293T packaging cells and used to infect cells in the presence of 8 µg/ml polybrene (Sigma H9268). Upon puromycin selection for 2–3 days, cells were returned to regular medium for experiments.

### Cell proliferation assay

Cells infected by lentivirus containing shGFP, shKu70 and shKu80 were seeded in triplicate in 24-well plates at 2500–5000 cells per well. Cells were fixed in 10% formalin and stained with 0.1% crystal violet on the day as indicated. Dye was extracted with 10% acetic acid and the proliferation was determined by measuring OD at 595 nm. Relative proliferation was calculated by normalization to day 0.

### Soft agar assay

2 mL of medium containing 1% agarose (Nobel Agar, BD 214230) was placed in 6-well plates as bottom layer. 2 mL of cell suspension with 5000 cells in medium containing 0.5% agarose was placed on top of solidified bottom layer. After 9–14 days, colonies were stained with p-iodonitrotetrazolium violet (Sigma, 18377), counted and photographed. If needed, NU7026 was added to both the bottom and top agar before being placed in the plates. 200 μL of medium containing inhibitors was added on the top every 3 days. For RNAi experiments, cells were seeded two days after transfection.

### Clonogenic survival assay

Cells were seeded in triplicate in 6-well plates at 100 cells/ well in growth medium, treated with NU7026 the next day, and radiated at 2, 4 and 6 Gy. After 10–14 day incubation, cells were fixed in 80% methanol and stained with 0.2% crystal violet and colonies were counted. The surviving fraction was calculated using the plating efficiency.

### Neutral comet assays

Neutral comet assays were performed according to the manufacturer's instructions (Trevigen). Briefly, cells were combined with low-melting agarose (catalog no. 4250–050–02), and then mounted on CometSlide (catalog no. 4250–200–03). Following cell lysis (catalog no. 4250–050–01) and unwinding of DNA, the cells were electrophoresed for 40 min at 21 volts in TBE buffer. Slides were fixed with ethanol, stained by SYBR and images taken by AutoComet machine (TriTek Corp). Minimal one-hundred randomly selected cells from each sample were analyzed using CometScore software (http://autocomet.com). DNA damage was determined by tail moment (tail length multiplied by the percentage of DNA in the tail).

### Western blot analysis

Cells were washed by PBS, counted and lysed in 2x SDS loading buffer. Western blot analysis was performed according to standard protocols. The following antibodies were used for Western: Actin (Sigma, A2066), γH2AX (Millipore, 05–636), Ku-70 goat (Santa Cruz, sc-1487), Phospho-Chk1 (S345) (cell signaling, #2348), and Cleaved caspase-3 (Asp175) (cell signaling, #9664).

### Immunofluorescent staining

Cells grown on coverslips or 8-well microscopy slides were fixed for 20 min with 4% paraformaldehyde and permeabilized by 0.1% Triton X-100 for 3 min. Cells were then incubated overnight at 4 degrees with mouse monoclonal antibodies against γH2AX (Millipore, 05–636), or Rad51 (Santa Cruz, sc-8349, H-92) followed by goat anti-mouse IgG-FITC (Santa Cruz, 1∶300) or goat anti-rabbit IgG-FITC (Santa Cruz, 1∶300). Cell images were taken under a Zeiss microscope using a 63x objective and analyzed for foci/nucleus.

### In vitro pGL2 plasmid –based NHEJ assay

pGL2-control plasmid (Promega) was completely linearized by the restriction endonuclease HindIII and the linearized DNA was extracted from agarose gel with Gel Extraction kit (Invitrogen). Circular plasmid and linearized DNA was then co-transfected with pRT-RL plasmid into cells with Lipofectamine 2000 (Invitrogen, 11668). The transfected cells were lysed and assayed for luciferase activity with Dual Luciferase Assay (Promega). The firefly signal was normalized to that of Renila which served as a transfection reference. Overall NHEJ capacity was calculated by firefly luciferase activity from cells transfected with HindIII-digested DNA relative to that of the intact plasmid.

### Flow cytometry assay

Cells were plated in 6-well plates, treated with NU7026 the following day for 48 hours, and radiated at 2 Gy and fixed after 24 hour with ice cold 70% ethanol in PBS for overnight. After centrifugation, pellets were resuspended in PBS, stained with propidium iodide solution for 30 min and analyzed by flow cytometry (BD FACS Calibur) in the dark.
